# Nano-engineering the titanium-tissue interface: a 15 year perspective on bio-functionalization and surface innovation

**DOI:** 10.1039/d6na00117c

**Published:** 2026-06-04

**Authors:** Aniruddha Vijay Savargaonkar, Ramesh Singh, Ketul C. Popat

**Affiliations:** a Department of Mechanical Engineering, Colorado State University Fort Collins CO 80523 USA kpopat@gmu.edu rsingh40@gmu.edu; b Department of Bioengineering, George Mason University Fairfax VA 22030 USA

## Abstract

Biofunctionalization of nanostructured titanium surfaces is a promising strategy to improve the performance of biomedical implants and devices. While titanium offers excellent mechanical properties and biocompatibility, it often encounters challenges such as bacterial infections, thrombogenicity, and complex cellular interactions. This review highlights decades of research in our laboratory on surface nanoengineering and biomaterial coating innovations that have significantly enhanced cellular adhesion, growth, and differentiation. Nanoengineered surfaces with adjustable wettability can significantly impact protein adsorption, cell adhesion, and blood compatibility. The potential of various biopolymer coatings, such as tanfloc, chitosan, heparin, and multilayer polyelectrolyte combinations, to promote beneficial cellular responses, support stem cell differentiation, and enhance hemocompatibility is also examined. Furthermore, the antimicrobial effects of these biopolymer-coated nanostructured titanium surfaces show promise in reducing infection risks associated with implants. Additionally, integrating small biomolecules, growth factors, and mineralization processes with relevant active metal ions reveals the potential of combining surface functionalization with nanoalteration. Finally, the review discusses current challenges and prospects in titanium implant surface engineering, underscoring the importance of further research to refine these technologies for therapeutic applications.

## Abstract 2

Biofunctionalization of nanostructured titanium surfaces is a promising strategy to improve the performance of biomedical implants and devices. This dual-modified interface enhances osseointegration, hemocompatibility, and bacterial resistance.

## Graphical abstract

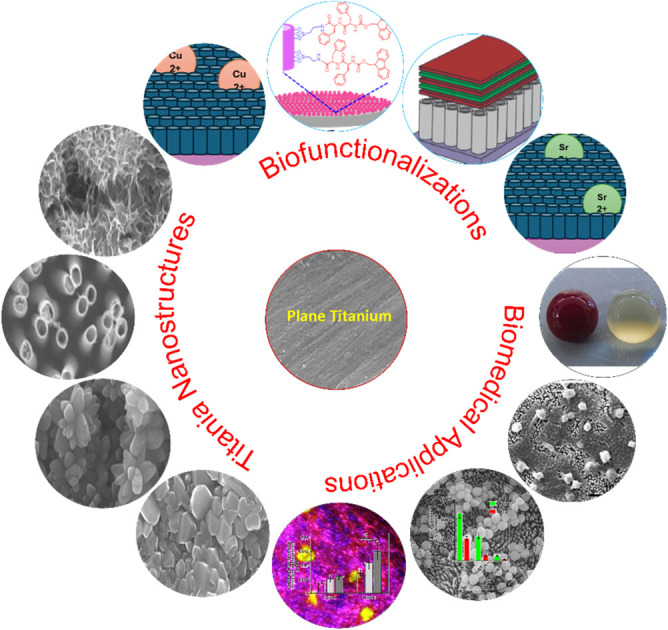

## 1. Introduction

Biomedical devices and implants are crucial in modern healthcare, addressing urgent health issues across various populations and regions. Technologies such as joint prosthetics that improve mobility and cardiac stents that prevent arterial blockages play vital roles in managing chronic illnesses, traumatic injuries, and age-related degenerative conditions.^1^ As global life expectancy increases and chronic diseases like arthritis, cardiovascular disorders, and osteoporosis become more common, the demand for innovative implant solutions continues to grow.^2^ These advancements enhance patients' quality of life and lower long-term healthcare costs by reducing complications, hospital stays, and the need for additional surgeries. The use of metal alloys in medical implants started in the 19th century, coinciding with progress in sterile surgical techniques. In the early 20th century, vanadium steel became the first metal alloy specifically designed for biocompatibility in human implantation.^3^

**Fig. 1:**
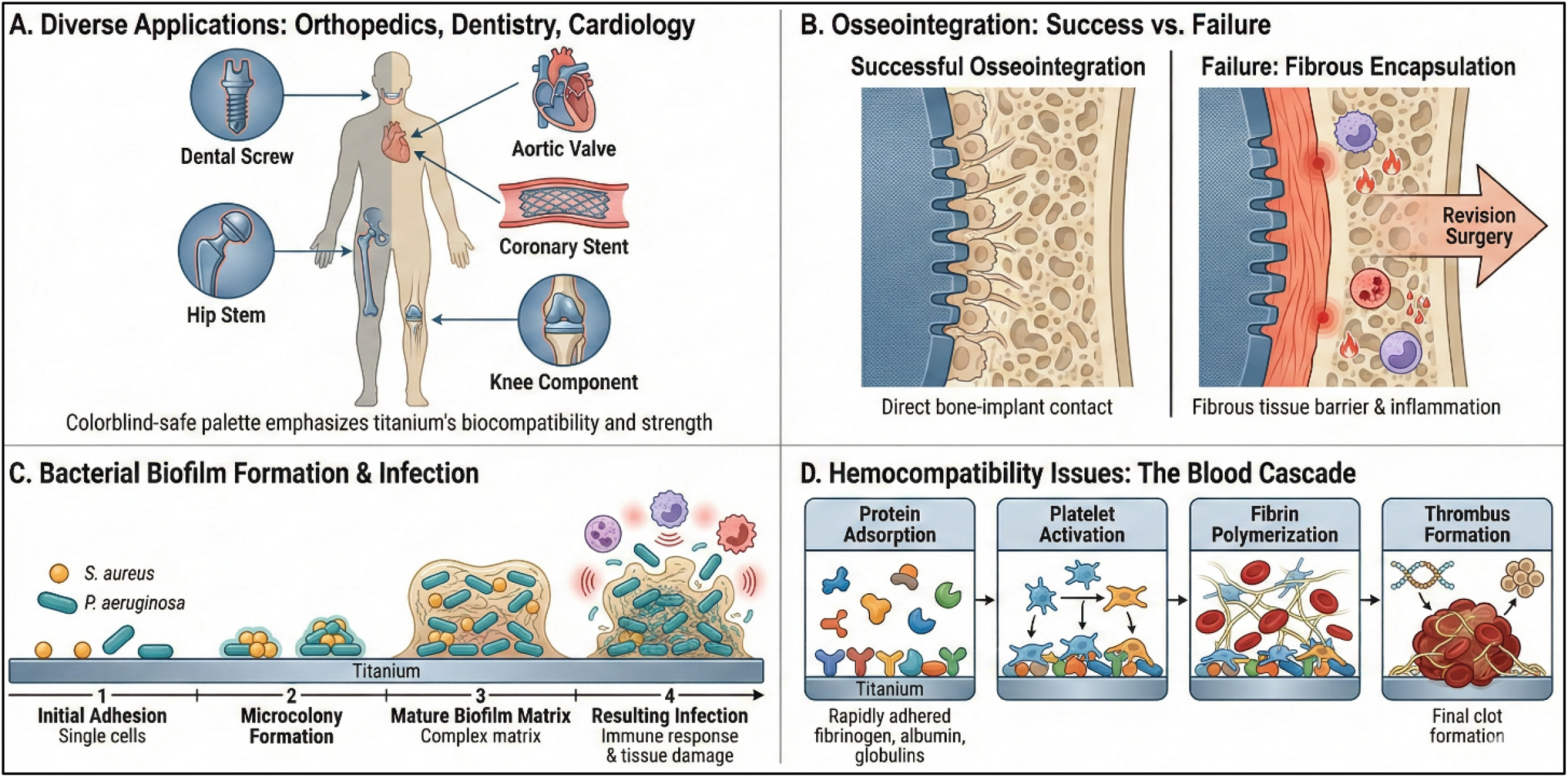
Schematic highlighting titanium-based implant applications and primary clinical failure modes. (A) Demonstrates widespread applications across various parts of the human body. (B) Depicts inadequate osseointegration resulting in fibrous tissue encapsulation and chronic inflammation.^9^ Structure redrawn from ref. 9 Copyright 2018, American Chemical Society (C) Bacterial biofilm progression leading to implant-associated infections.^10^ Reproduced with permission from ref. 10. Copyright 2020, Elsevier (D) Shows the blood clotting cascade initiated on biomaterial surfaces, beginning with plasma protein adsorption, followed by platelet adhesion and activation, cellular deposition, fibrin polymerization, and eventual thrombus formation.^11^ Reproduced with permission from ref. 11 Copyright 2021, Royal Society of Chemistry.

Modern implants primarily rely on titanium-based materials due to their exceptional mechanical strength, corrosion resistance, and biocompatibility, making them popular choices for orthopedic and dental implants (Fig. 1).^4^ However, the clinical performance of titanium implants faces several issues, including poor osseointegration and vulnerability to bacterial growth, which can lead to implant failure.^5^ For example, bacteria on titanium surfaces can cause implant-related infections, while inadequate osseointegration may result in implant failure (Fig. 1). Likewise, biofouling, the accumulation of proteins and cells on the implant surface, can lead to inflammation and impair device function.^5^ A lack of proper integration can lead to the formation of fibrous tissue, which may loosen the implant and necessitate revision surgery.^6,7^ In the USA, 37 000 out of every million hip and knee replacement surgeries are revision procedures.^8^ These challenges highlight the need for new strategies to improve the biological performance of titanium-based Implant materials.

The biological response to implants is mainly influenced by the surface properties of biomaterials, such as structural topography, chemical composition, and surface characteristics energy.^12^ These properties regulate vital processes, including cell adhesion dynamics, protein deposition patterns, and immunological activation, which collectively influence outcomes such as bone integration, microbial colonization, and inflammation responses.^13^ For example, nanoscale-textured surfaces replicate the natural architecture of bone tissue, promoting osteogenic cell attachment and supporting bone repair mechanisms.^14^ Moderately hydrophilic surfaces promote enhanced cellular growth while supporting anti-inflammatory immune cell behavior. In contrast, hydrophobic surfaces or those with suboptimal microstructural features reduce cell attachment and increase antifouling activity.^8,15^ This was the focus of second-generation biomaterials. This new generation of biomaterials emphasizes increasing bioactivity and the material's ability to interact with surrounding tissue, thereby establishing a direct bond, compared to the older generation, which was focused on being inert. As surface interactions are critical in determining biological response, surface engineering can enhance the bioactivity of materials.

Modern surface-engineering innovations have enabled new solutions to challenges related to titanium implants. For instance, titanium surfaces engineered with nanoarchitectures, such as TiO_2_ nanotube arrays, mimic the nanoscale features of natural bone tissue, thereby promoting better biological integration responses.^16,17^ These nanostructured interfaces enhance osseointegration by promoting bone cell attachment and growth, while also exhibiting antimicrobial effects through mechanisms such as physical disruption of bacterial membranes or localized delivery of therapeutic agents.^18–20^ Further enhancements are achieved through functional polymer coatings, which offer benefits such as reduced biofouling, controlled drug release, and improved performance and biocompatibility.^21,22^ Materials such as biopolymers, biomolecules, growth factors, and metal ions effectively reduce unwanted protein adhesion, inhibit microbial growth, and enable targeted therapy at implantation sites. Biopolymer coatings provide an additional method of surface functionalization. These polymer-based coatings, whether synthetic or natural, can be engineered to deliver therapeutic materials, provide specific biological cues, or enhance the interface between the surrounding tissue and the implant.^23^ The strategic integration of nano topography with multifunctional polymeric layers creates versatile platforms capable of meeting a wide range of clinical needs, paving the way for next-generation implant technologies with enhanced safety and effectiveness.

This review primarily examines our group's 15 years of research on the functionalization of titanium surfaces for biomedical applications. We focus on two complementary biofunctionalization strategies: the creation of nanostructured titanium surfaces through methods such as plasma electrolytic oxidation and chemical etching, and the application of surface coatings, including polymer layers and nanomaterial-based modifications. By combining nanoscale topography with bioactive chemistry, these approaches aim to improve implant performance by modulating bacterial adhesion, tissue integration, hemocompatibility, and cellular response. The review critically discusses the individual and combined roles of these strategies in orthopedic, vascular, and dental applications, while also highlighting current limitations, translational barriers, and future opportunities for advancing titanium-based biomedical technologies.

## 2. Engineering titania nanostructures on the titanium surface

Nanostructures are materials or objects whose at least one dimension in the nanometer range falls between molecules and microscopic structures, typically ranging from 1 to 100 nanometers.^24^ Nanostructured surfaces are generally described as one, two, or three dimensions in the nanoscale.^25^ Their unique size-dependent properties have made them highly significant in the biomedical field, driving advancements in diagnostics, therapeutics, and regenerative medicine. For example, nanostructures such as nanoparticles, nanofibers, and nanotubes are utilized for targeted drug delivery, enabling the transport and controlled release of therapeutic agents directly to diseased cells, thereby minimizing systemic side effects and improving treatment efficacy.^26^ In tissue engineering, nanostructured scaffolds mimic the natural extracellular matrix, promoting cell adhesion and tissue regeneration.^27^

Incorporating nanostructures onto biomedical implants provides numerous significant advantages, significantly improving their functionality and clinical outcomes. Fabrication of nanostructures on biomaterial surfaces changes the surface topography, roughness, and wettability. These properties promote a more harmonious interaction between the implant and surrounding biological tissues, thereby significantly enhancing biocompatibility and reducing immune rejection and side effects.^31,32^ Nanostructures on implant biomaterials can reduce bacterial adhesion and growth by disrupting bacterial cell membranes.^16,19,33^ Nanostructured implant surfaces can promote osseointegration by supporting the adhesion, proliferation, and differentiation of mesenchymal stem cells (MSCs) or bone cells, thereby enhancing tissue integration and regeneration, and speeding up healing processes by accommodating the mechanical stress generated by the cells.^34^

**Fig. 2:**
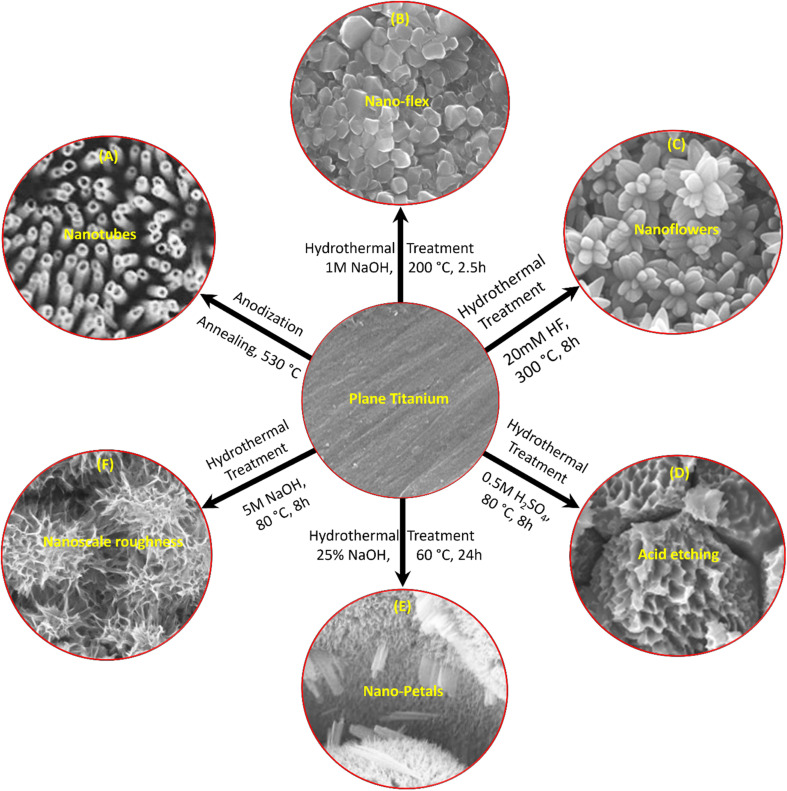
Various modifications of TiO_2_ nanostructures on titanium surfaces are designed to improve surface topographical cues for enhanced biointerface performance. (A) Formation of highly ordered nanotubes through anodization of titanium in an electrolyte solution (DEG + HF + DI), followed by thermal annealing in an oxygen environment.^7^ Adapted with permission from ref. 7. Copyright 2024, Royal Society of Chemistry. (B) Synthesis of nano-flex structures through hydrothermal treatment in an alkaline environment, followed by annealing and another hydrothermal process using HCl.^28^ Adapted with permission from ref. 28. Copyright 2020, American Chemical Society. (C) Nanoflower morphologies produced through hydrothermal treatment in an acidic environment medium.^29^ Adapted with permission from ref. 29. Copyright 2021, Elsevier. (D) Acid-etched surfaces were created through a hydrothermal treatment process, followed by annealing.^30^ Adapted with permission from ref. 30. Copyright 2024, Springer Nature. (E) Nano-petal structures were produced using hydrothermal treatment with 25% NaOH, followed by annealing.^14^ Adapted with permission from ref. 14. Copyright 2025, MDPI. (F) Generation of nanoscale roughness through hydrothermal treatment using 5 M NaOH.^28^ Adapted with permission from ref. 28. Copyright 2020, American Chemical Society.

Various nanostructures have been observed on the surfaces of titanium biomedical implant devices, including nanofibers, nanopores, nanoflakes or nanopetals, nanowires, nanotubes, and nanoflowers (Fig. 2).^7,29,35–37^ Each is designed to enhance specific biological responses and implant performance (Table 1). Nano-structuring titanium surfaces for biomedical implants involves several advanced fabrication techniques. Electrochemical anodization is a common method for producing well-ordered titanium dioxide (TiO_2_) nanotubes and nanopores by applying a voltage in a fluoride-containing electrolyte. This enables precise control over nanotube size and arrangement.^22,36^ Nanoflakes or nanopetals are formed through hydrothermal and alkali-heat treatments, which involve exposing the metal to concentrated alkaline solutions at high temperatures and pressures.^14^ The resulting surface characteristics depend on the specific treatment conditions. Aside from these two main methods, laser-based techniques, including direct laser writing and laser interference patterning, enable the creation of highly controlled and hierarchical nanoscale patterns on titanium surfaces. Additionally, chemical etching with acids or alkalis provides a simple method for generating nano- and microscale roughness on titanium surfaces (Fig. 2).

**Table 1:** Different types of nanostructures are fabricated on titanium surfaces

Nanostructure	Technique	Primary reagent(s)	Ref.
Nanotubes	Anodization	Diethylene glycol (DEG), hydrofluoric acid (HF), deionized water (DI)	38
Nanoflowers	Hydrothermal treatment	Deionized water (DI) & hydrofluoric acid (HF)	29
Nanopores	Anodization	Oxalic acid, chromic acid, and phosphoric acid	36
Nanoflakes or nanopetals	Hydrothermal treatment	Deionized water (DI) & sodium hydroxide (NaOH)	14 and 28

Among all these nano-alterations, titania (TiO_2_) nanotubes (NTs) have been a major focus of research in our laboratory, especially those created through electrochemical anodization. This method involves oxidizing the titanium surface and etching it in an electrolyte containing hydrofluoric acid (HF) and diethylene glycol (DEG), resulting in the formation of nanotube arrays.^7,32^ The structural features of these nanotubes, such as their diameter, length, and overall shape, can be precisely controlled by modifying the electrolyte composition, the applied voltage, and the anodization duration. For example, using an electrolyte made of 99% DEG, 2% HF (48% v/v), and 3% deionized water, NTs with diameters of 110 nm can be generated at 45 V. Increasing the voltage to 60 V results in diameters of 160 nm after 24 hours, while lowering the voltage to 30 V produces nanotubes with a diameter of 70 nm.^39^ DEG, as an organic solvent, helps manage the viscosity and dielectric properties of the electrolyte, leading to smoother, more uniform, and longer nanotubes than those formed in purely aqueous HF solutions.^40^ When cleaned, titanium surfaces were anodized with a platinum cathode at 55 V in an electrolyte solution of 2% HF (48% v/v), 95% DEG, and 3% DI water for 22 hours; the resulting NTs typically have diameters ranging from 70 to 100 nm.^41^ Research indicates that NTs with diameters of 70–80 nm are particularly effective at inhibiting the growth of periodontal pathogens and can help prevent early biofilm formation.^42^ Larger nanotube diameters (80–120 nm) have been shown to promote bone formation (osteogenesis) while reducing the activity of bone-resorbing cells (osteoclasts).^39^

## 3. Biofunctionalization of nanostructured titanium surfaces: combined impact of nanotopography and functional biomaterials

A sophisticated combination of structural and biochemical environments is responsible for natural bone growth, supporting its mechanical function, cellular activity, and biochemical processes. The natural bone environment is defined by a mineralized collagenous matrix that balances rigidity (from minerals) and flexibility (from collagen), supporting both mechanical function and biological activity.^43^ The extracellular matrix (ECM) is a dynamic structure that is constantly remodeled by osteoblasts and osteoclasts, serving as a reservoir for growth factors and minerals essential for bone homeostasis and regeneration.^44^ The interplay of structure and chemistry in natural bone creates an optimal environment for cell attachment, proliferation, differentiation, and vascularization, critical for bone growth and repair.

To mimic the natural environment of bone growth, biomaterials must exhibit both structural and chemical properties that closely resemble those of native bone.^45^ Structurally, a porous architecture with interconnected pores at the micro- and nanoscale that supports cell infiltration, vascularization, and nutrient exchange is similar to natural bone tissue.^14^ Chemically, biomaterials must be biocompatible and bioactive, incorporating components such as hydroxyapatite or calcium phosphate to emulate the mineral phase of bone, and natural or synthetic polymers (*e.g.*, collagen, chitosan, polycaprolactone) to mimic the organic matrix and promote cell adhesion and differentiation.^46^ Additional features such as controlled degradability, osteogenicity, and the ability to deliver bioactive molecules or drugs further enhance their capacity to create a bone-like micro-nano-environment that supports stem cell recruitment, angiogenesis, and new tissue formation.^47^

Nanostructured titania surfaces provide a more biomimetic environment than standard titanium, leading to increased osteoblast adhesion, enhanced protein interactions, and more robust cellular signaling.^48^ These features contribute to higher rates of cell proliferation. The nanoscale topography of these surfaces closely resembles the natural extracellular matrix, which encourages the selective attachment and spreading of osteoblasts and other bone cells.^49^ Additionally, nanostructures increase the effective surface area, surface roughness, and hydrophilicity, allowing enhanced adsorption of proteins that mediate cell attachment and signaling and lead to improved cell proliferation.^50,51^ On the other hand, biomaterials such as biopolymers, hydroxyapatite, or biomolecules are known to serve as chemical scaffolds that support cell adhesion, proliferation, and differentiation, closely mimicking the natural extracellular matrix and facilitating tissue integration and regeneration.^52^ Due to their excellent biocompatibility, biopolymers help reduce the risk of immune rejection and inflammation, making them suitable for direct contact with body tissues. Their biodegradability allows for temporary implants that gradually degrade as new tissue forms, eliminating the need for surgical removal.^52^ Additionally, these polymers have the potential to be functionalized for local delivery of therapeutic agents, such as antibiotics or growth factors, thereby enhancing healing and reducing systemic side effects.^53^

**Fig. 3:**
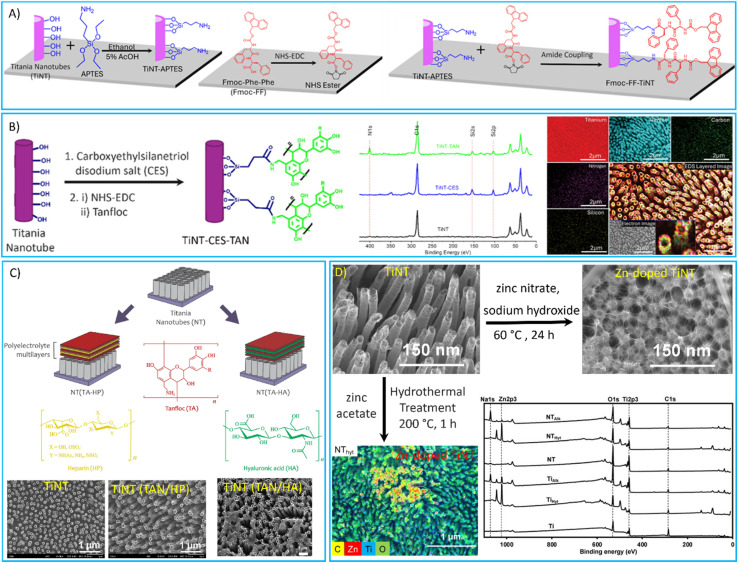
Representative examples of various surface coating strategies applied to titanium-based substrates to enhance their biofunctionality. (A) Covalent conjugation of Fmoc-FF dipeptides onto titania nanotube (TiNT) surfaces using silane chemistry.^54^ Reproduced with permission from ref. 54. Copyright 2024, American Chemical Society. (B) Surface modification of TiNTs using tanfloc *via* covalent bonding and characterization results, XPS, and color map EDS of the modified surfaces.^48^ Reproduced with permission from ref. 48. Copyright 2025, American Chemical Society. (C) Deposition of tanfloc–heparin and tanfloc–hyaluronic acid polyelectrolyte multilayers (PEMs) on TiNT surfaces, with morphological evaluation using electron microscopy.^6^ Reproduced with permission from ref. 6. Copyright 2021, Elsevier. (D) SEM images demonstrate the morphology of pristine TiNT and zinc-doped TiNT (Zn-TiNT) fabricated *via* a hydrothermal method, and EDS and XPS analyses confirm the presence and distribution of zinc.^20^ Reproduced with permission from ref. 20. Copyright 2023, MDPI.

In the broader field of titanium implant surface engineering, several approaches have been explored, including TiO_2_ nanotube fabrication, plasma electrolytic oxidation, chemical etching, hydroxyapatite coatings, ion-doped surfaces, antimicrobial peptide coatings, biopolymers, and drug-delivery-based functional layers. These strategies differ in fabrication complexity, coating stability, biological performance, and translational readiness. Our group has focused on integrating the advantages of nanostructured surfaces with the functional benefits of biopolymers and biologically active molecules. This dual modification of the titanium surface creates a synergistic effect that significantly enhances the biological integration of the implant and reduces the risk of failure (Fig. 3). For example, titania nanotube arrays, with their corrosion resistance, surface stability, and tunable dimensions, provide structural cues that resemble the native bone microenvironment. Their large surface area and strong surface adhesion can enhance protein adsorption and support the adhesion, proliferation, and differentiation of bone cells. When combined with bioactive materials such as polymers, hydroxyapatite, or biomolecules, they further enhance biocompatibility, reduce cytotoxicity, and provide biological signals for bone regeneration. Such biofunctionalization can also contribute to antibacterial and anti-inflammatory effects, controlled drug release, and preservation of mechanical stability, thereby supporting osseointegration, sustained therapeutic delivery, and reduced infection risk (Fig. 3).

Table 2 lists biomaterials and drugs used to functionalize nanostructured titania surfaces. In addition to commonly used natural biopolymers, we employed an amino-functionalized tannin derivative, tanfloc, to modify titania nanotube surfaces, both alone and in combination with other polymeric electrolytes (Fig. 3). Tanfloc is a versatile biopolymer featuring polyphenolic, amino, and aromatic groups, and it exhibits amphiphilic properties as both a polycationic and polyanionic substance (Fig. 3B and C). Its amphoteric properties enable tanfloc to interact with various counter-polyelectrolytes, forming stable complexes that enhance the stability of coatings on biomaterials. Research shows that tanfloc, whether used alone or in combination with polyelectrolytes such as heparin, glycosaminoglycans, alginate, and chitosan, demonstrates significant biocompatibility, antibacterial properties, and the ability to promote osteogenic differentiation with minimal cytotoxicity.^48,55^

**Table 2:** Different coatings deposited on titanium and titania nanostructures

Bio-functionalization with	Nanostructure surface	Technique used	Application	Ref.
**Biopolymers**				
Tanfloc	Nanotubes	Covalent	Antibacterial activity	21
Tanfloc/heparin	Nanotubes	PEM	Antibacterial activity	22 and 55
			Superhemophobic	
Tannin/glycosaminoglycan	Nanotubes	PEM	Stem cells' adhesion and proliferation endothelialization nanostructured	56
BMP-2 with chitosan/heparin	Nanotubes	PEM	Increased osteoblast differentiation	57

**Small organic molecules and drugs**				
Fmoc-Phe-Phe	Nanotubes	Covalent	Antibacterial activity	54
Gentamicin	Nanotubes	Lyophilization	Antibacterial activity	58
			Increased osteoblast differentiation	
(Heptadecafluoro-1,1,2,2-tetrahydrodecyl)trichlorosilane	Nanotubes	Chemical vapor deposition	Antibacterial activity	15 and 59–61
	Nanoflowers		Superhemophobic	
	Nanostructured surfaces			
Octadecyltrichlorosilane	Nanotube	Chemical vapor deposition	Superhemophobic	60

**Matelic ions**				
Copper	Nanotubes	Physical vapor deposition	Antibacterial activity	7 and 19
			Increased osteoblast differentiation	
Zinc	Nanotubes	Hydrothermal treatment	Antibacterial activity	20 and 34
		Alkaline heat-treatment	Increased osteoblast differentiation	
Strontium	Nanotubes	Hydrothermal	Increased osteoblast differentiation	34
Silver	Titanium	Sputter deposition	Antibacterial activity	62
Calcium and phosphorus	Nanotubes	Anodization process	Increased cell adhesion and proliferation	63

Titania nanotubes, along with these PEMs, can be used for therapeutic delivery. For chitosan/heparin-based PEM used for bone morphogenetic protein-2 (BMP-2). To further enhance the stability of polymer functionalization, a covalent grafting strategy was employed for longer-term results. Different types of hydrophilic nanostructures, such as nanoflowers, nanotubes, and hydrothermally modified titanium surfaces, were converted into hydrophobic surfaces through salinization. Heptadecafluoro-1,1,2,2-tetrahydrodecyl trichlorosilane, poly-ethyleneglycol 2-[methoxy(polyethyleneoxy)propyl] trimethoxy-silane, and (heptadecafluoro-1,1,2,2-tetrahydrodecyl) trichlorosilane, *etc.*, were used to modify the wettability of hydrophilic nanostructured surfaces.^15,29,59^

## 4. Incorporation of biologically and clinically significant metal ions

Bone is a mineralized form of connective tissue composed of bone cells, several bioactive substances, and an extracellular matrix with textures at the nano and microscales.^64^ Trace metals, such as strontium, zinc, copper, and silicon, play a crucial role in bone development, bone metabolism, and other physiological functions. Therefore, various metals, including silver (Ag), copper (Cu), manganese (Mn), strontium (Sr), and zinc (Zn), have been utilized to coat titania nanotube surfaces for different functionalities.^7,22,34,62,65^ For example, Ag and Zn are well known for their antibacterial activity, and are used for enhancing the antibacterial activity of the surfaces.^20,62^ On the other hand, metals such as Cu play a significant role in bone regeneration either directly or indirectly by modulating the inflammatory responses, oxidative stress, and rapamycin signaling. Copper ions released from biological materials can affect osteoblasts and osteoclasts, promoting bacterial killing when they come into contact.^19^

We also utilized the physicochemical properties of these biologically relevant metals, including Sr, Cu, Zn, and Ag, which were deposited over nanostructured titania surfaces (Table 2). These modified nanostructured titanium surfaces enhance their biocompatibility and antibacterial activity. One-step physical vapor deposition, hydrothermal treatment, and sputter deposition techniques were mainly used for metal deposition. The titania nanotube surfaces were subjected to Cu deposition at about 5 × 10^−5^ Torr base vacuum, followed by annealing to promote Cu diffusion.^19^ However, Zn and Sr deposition over titania nanotubes was achieved by heating in a hydrothermal autoclave reactor at 200 °C.^20^ Ag deposition was achieved *via* ion-beam sputtering. Titanium surfaces were cleaned and plasma-etched, followed by sputtering of Ag and hydroxyapatite.^62^ Furthermore, in a study, manganese is used in combination with bioactive glass to functionalize titania nanotubes, thereby enhancing the surface biocompatibility.^65^

## 5. Biomedical outcomes of biofunctionalized nanostructured surfaces

Surface modifications are implemented to achieve favorable surface properties, which in turn enable favorable outcomes. The work conducted by the Popat lab is focused on achieving the following outcomes: antibacterial activity, hemocompatibility, and modulation of the cellular response. In this section, we will focus on the functionalization achieved by different surface modifications (nanostructured engineering and/or biomaterial coatings) when interacting with bacteria, blood, and human cells.

### 5.1 Advancing in antibacterial activity

**Fig. 4:**
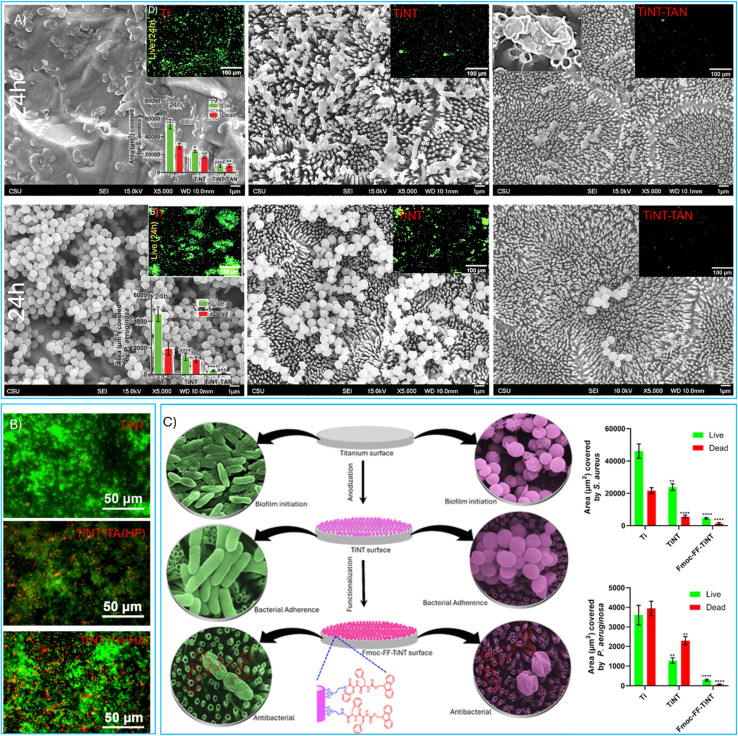
Improved antibacterial performance of titanium surfaces through various surface modification strategies. (A) Representative SEM images depicting bacterial adhesion on unmodified Ti, TiNT, and TiNT-TAN after 24 hours of incubation. Inset fluorescence images and bar graphs quantify surface area coverage by live (green) and dead (red) bacteria.^21^ Reproduced with permission from ref. 21. Copyright 2024, Wiley. (B) Fluorescence microscopy images showing *Staphylococcus aureus* colonization on TiNT, and modified surfaces, with tanfloc–heparin (TA/HP) and tanfloc–hyaluronic acid (TA/HA), PEM coatings after 24 hours.^22^ Reproduced with permission from ref. 22. Copyright 2020, Wiley. (C) Results from a nanotubular titanium surface functionalized with an antibacterial dipeptide, against *Pseudomonas aeruginosa* and *Staphylococcus aureus* after 24 hours of exposure.^54^ Reproduced with permission from ref. 54. Copyright 2024, American Chemical Society.

Post-operative bacterial infections are the most dreaded complications of medical implants, often leading to implant failure.^66^ Treating these infections in orthopedic implants requires reoperation on the implant, a series of procedures that increase healthcare costs, prolong treatment, and are sometimes unsuccessful.^67^ When bacteria adhere to the surface of orthopedic implants, they form a self-protective layer of proteins called biofilm, rendering them immune to antibiotics. Implants can become a haven for opportunistic bacteria, such as *Staphylococcus aureus*, *Staphylococcus epidermidis*, and *Pseudomonas aeruginosa*, which are otherwise not highly virulent but can evade host defenses and antibiotic therapy once they colonize the implant surface (Fig. 4).^55,68^

The first step in preventing bacterial infection is to prevent bacterial adherence and inhibit biofilm formation.^69^ Nano-structuring alters the topography of the titanium surface, making it more difficult for bacteria to adhere compared to unmodified titanium.^59^ The fabrication of nanostructures, such as titania nanotubes and nanoflowers, and the application of hydrothermal treatment to the titanium surface have enhanced antibacterial activity at an early stage compared to unmodified titanium.^8,29,58^ For example, early contact of bacteria such as *Pseudomonas aeruginosa* with a larger area led to membrane disruption by the titania nanotubes, a phenomenon known as contact killing.^70^ Additionally, these nanotubular surfaces have exceptional loading capacity, and titania nanotube surfaces loaded with antibacterial drugs like gentamicin enhance their antibacterial effect.^18,58^

Tanfloc- or tannin-based PEMs functionalization to create an antimicrobial coating by combining them with polyanionic polysaccharides such as heparin, hyaluronic acid, chondroitin sulfate, pectin, and iota-carrageenan.^55^ PEMs formed from chitosan or tanfloc in combination with these polyanions exhibit both antiadhesive and antimicrobial effects, effectively minimizing bacterial attachment and growth on treated surfaces.^71^ The application of PEMs aims to inhibit bacterial attachment and colonization, particularly during the initial days after implantation, thereby reducing the risk of biofilm development.^71^ It was found that titania nanotubular topography, together with tanfloc/heparin and tanfloc/hyaluronic acid PEMs functionalization, reduces bacterial adhesion and proliferation. This combination was tested against both Gram-positive and Gram-negative infection-causing bacteria, *Staphylococcus aureus* and *Pseudomonas aeruginosa*. TiNT-tanfloc/heparin surfaces effectively inhibited *Pseudomonas aeruginosa* (Fig. 4B).^22^

Further, the antibacterial activity of only tanfloc with titania was also investigated. Covalently attached tanfloc on TiNT surfaces was subjected to test against *Staphylococcus aureus* and *Pseudomonas aeruginosa*.^21^ Interestingly, these surfaces demonstrated effective antiadhesive and antibacterial properties against both Gram-positive and Gram-negative bacteria.^21^ This anti-adhesive and antibacterial activity can be attributed to the amphoteric or zwitterion-like nature of tanfloc, which allows it to repel bacterial adhesion to the surface.^21^ Additionally, the multifunctionality of these surfaces, including topography and tanfloc chemistry, including polyphenolic and positively charged ammonium groups, electrostatically interacts with the negatively charged bacterial membrane and disrupts it. Recently, we developed covalently conjugated self-assembling short peptides on the titania nanotube surface, which increases the antibacterial properties.^72^ Strategically designed short peptides exhibit a multifaceted mechanism of action to inhibit bacterial infections.^73,74^ They interact with the bacterial cell wall and disrupt it, resulting in bacterial death.^73–75^ Fmoc-protected diphenylalanine (Fmoc-FF) peptides have been found to trigger both oxidative and osmotic stress in bacterial cells, thereby increasing their antibacterial activity.^76^ Fmoc-FF modified surfaces demonstrate a significant reduction of attachment and impeded biofilm development (Fig. 4C).

Additionally, doping of titania nanostructures with transition metals like silver (Ag), zinc (Zn), and copper (Cu), having inherent antibacterial properties, has also been studied, and found that the trace amount of these metal ions doped in titania nanotubes significantly increases the contact killing of bacterial cells^19^ and exhibits antibacterial activity against both Gram-positive and Gram-negative bacteria.^20,62^ Metal functionalized titania nanotube surface enables the controlled release of metal cations that can penetrate bacterial membranes, disrupt metabolic processes, and generate reactive oxygen species (ROS), all of which contribute to bacterial cell death.

### 5.2 Optimization of blood and surface interactions

Hemocompatibility is another essential factor in determining the biocompatibility and success of medical implant devices. However, hemocompatibility is not a well-defined term and varies considerably depending on the type of device or application. It can be broadly described as the ability of a biomaterial to interact with blood without causing adverse effects such as thrombus formation or hemolysis.^77^ Whenever blood comes into contact with foreign elements, biomaterials, or medical devices, a series of interactions is immediately triggered. It begins with protein adsorption, inflammation, and hemolysis, followed by platelet activation and/or fibrosis, which lead to blood coagulation or thrombus formation.^41^ Blood coagulation can be either beneficial or harmful, depending on the purpose of the medical implant. For example, platelet activation provides advantages in orthopedic surgeries such as joint replacements, spinal fusions, and bone-grafting procedures.^7^ However, in cardiovascular treatments, excessive platelet-driven clot formation can lead to serious complications, including blocked blood vessels, aneurysms, or even cardiac issues arrest.^60^ Since the implant surface initiates all these interactions and depends on various material properties, such as surface chemistry, topography, wettability, charge, and porosity. Therefore, it is essential for medical devices to function in a controlled manner that responds intelligently and effectively, regulating blood clotting to encourage healing while minimizing the risk of harmful thrombosis.^78^

Engineering titania surfaces with nanostructures like nanotubes, nanoflowers, and nanopetals results in a consistent surface texture and enhances hydrophilicity. Increased hydrophilicity promotes the attachment and spreading of water molecules, thereby decreasing fibrinogen adsorption. Since fibrinogen adsorption activates the intrinsic blood coagulation pathway, elevated levels of surface-bound fibrinogen can speed up clot formation and, in certain cases, contribute to thrombosis. Therefore, the nanostructured surface decreases fibrinogen adsorption, thereby lowering platelet activation compared to planar titanium.^28,41^ Platelet activation is a crucial step in blood clotting and tissue repair. Platelet activation produces alpha granules that contain numerous growth factors, creating a scaffold for tissue repair and preventing blood loss.^48^

**Fig. 5:**
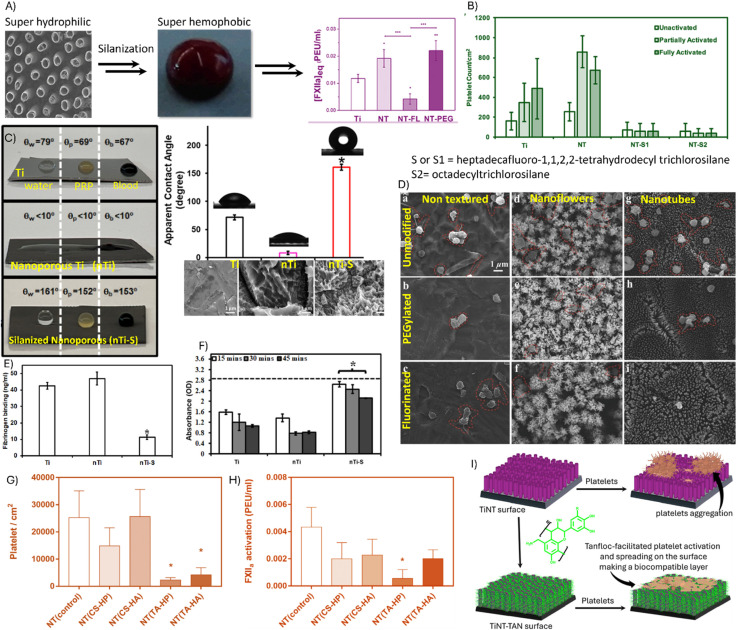
Overview of various strategies for modulating hemocompatibility on titania-based surfaces *via* surface chemistry and topographical modifications. (A) Surface wettability tuning of titania nanotubes using silane modification, transitioning from super-hemophilic to super-hemophobic, resulting in a decrease in adherence of blood components.^79^ Reproduced with permission from ref. 79. Copyright 2019, Elsevier. (B) Quantification of platelet adhesion on different titanium surfaces: plain titanium (Ti), unmodified titania nanotubes (NT), and NT modified silanes.^60^ Reproduced with permission from ref. 60. Copyright 2017, Royal Society of Chemistry. (C) Optical image showing the interaction and apparent contact angle measurements of different surfaces with Milli-Q water, PRP, and blood, and representative SEM images.^59,80^ Reproduced with permission from ref. 59 and 80. Copyright 2022, MDPI and 2022, Wiley. (D) SEM images highlight platelet activation (indicated by red dotted outlines) on titania surfaces with different chemical modifications: unmodified, PEGylated, and fluorinated non-textured surfaces.^81^ Reproduced from ref. 81. Copyright 2016, Wiley. (E) Fibrinogen adsorption from PRP.^80^ (F) Whole blood clotting kinetics on various surfaces over a 45 minutes period. The dotted line indicates the absorbance corresponding to free hemoglobin in unclotted blood.^80^ Reproduced with permission from ref. 80. Copyright 2022, MDPI. (G) Quantification of platelets adherent (per cm^2^) PEM modified surfaces.^22^ (H) FXII activation on different surfaces.^22^ Reproduced with permission from ref. 22. Copyright 2020, Wiley. (I) Schematic showing modification of titania nanotube surfaces with tanfloc and the effect it has on the platelet adhesion and activation.^48^ Reproduced with permission from ref. 48. Copyright 2025, American Chemical Society.

For example, titania nanotube surfaces, which are hydrophilic, showed reduced platelet activation compared with bare titanium surfaces. When these nanotubular surfaces were doped with metallic ions such as copper and zinc, the hydrophilicity increased further, leading to a greater decrease in fibrinogen adsorption compared to bare titanium and subsequently decreasing platelet adherence and activation. The titania nanoflowers also exhibit superhydrophilic properties and lower platelet adhesion than the unmodified titanium surface.^29^ Similarly, hydrothermally treated surfaces generated web-like nanoporous, hydrophilic titania surfaces, which demonstrated a significant decrease in platelet adhesion (Fig. 5).^14,28^

Combining titania nanotubes with layers of the biopolymers tanfloc and heparin has been shown to reduce fibrinogen adsorption and significantly lower FXII activation.^22^ This modification also results in decreased platelet adhesion compared to unmodified nanotubes. In more recent work, it was found that when tanfloc is covalently attached to titania nanotubes alone, platelet activation is altered, yet fibrinogen adsorption remains similarly reduced. Tanfloc-modified nanotubes still exhibit reduced platelet adhesion, but platelets are more evenly distributed and spread across the surface, rather than forming aggregates as seen on unmodified nanotubes.^48^ Interestingly, the observation of low fibrinogen adsorption alongside pronounced platelet activation on TiNT-Tanfloc surfaces suggests that tanfloc directly affects platelet behavior, rather than acting solely through fibrinogen-mediated pathways. Tanfloc, as an amphoteric polymer with both polycationic and polyanionic properties, displays zwitterionic-like behavior, enabling it to neutralize charges. Unlike conventional zwitterionic polymers that completely inhibit platelet activation, tanfloc may facilitate more selective and controlled interactions with blood proteins and platelets (Fig. 5).

Generally, surfaces with titania nanostructures are hydrophilic, unlike the superhydrophobic titanium surface. Surface wettability plays a key role in the performance and integration of implant devices, affecting biological interactions at the implant–host interface. Carefully controlling surface wettability is essential for medical devices used for various purposes. Converting the surface to super-repellent is an effective way to achieve highly hydrophobic or hemophobic properties. This change significantly reduces the contact area between blood and the material, limiting where proteins, blood cells, or platelets can adhere. Additionally, super-repellent surfaces promote slip at the interface and alter shear stresses, which can help reduce damage to blood cells and platelets. For example, the chemical vapor deposition of (heptadecafluoro-1,1,2,2-tetrahydrodecyl) trichlorosilane on titania nanostructures, such as nanotubes and nanoflower surfaces, converts them into superhydrophobic surfaces.^29^ Findings showed that these superhemophobic surfaces had lower adsorption of albumin, fibrinogen, and factor XII, compared to unmodified surfaces. Additionally, significant decreases in PRP cell adhesion and platelet activation were observed.^29,61^

### 5.3 Advances in cell–surface interactions

**Fig. 6:**
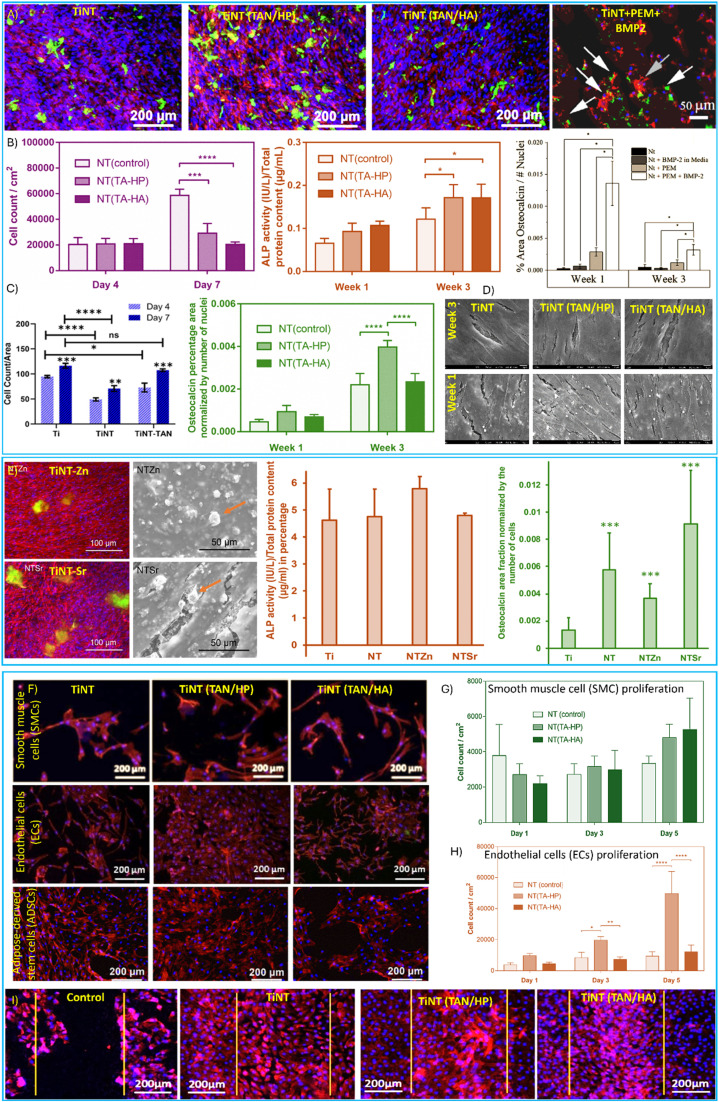
Representative images and quantitative analyses highlighting the cellular responses of various biofunctionalized titanium nanotube (TiNT) surfaces. (A) Immunofluorescence microscopy images of ADSCs after 3 weeks of osteogenic induction, showing green fluorescence for osteocalcin expression on different TiNT surfaces: unmodified TiNT and TiNT with (TA/HP), (TA/HA), and chitosan–heparin, along with BMP-2 loading.^6,57^ (B) Quantification of ADSC adhesion and proliferation on PEM modified TiNT, TiNT(TA/HP), and TiNT(TA/HA) surfaces^6^ after 4 and 7 days (left); ALP activity normalized to protein content after 1 and 3 weeks (middle); and % area of osteocalcin per nucleus after 1 and 3 weeks on TiNT, TiNT + PEM, TiNT + BMP2, and TiNT + PEM + BMP2 (right).^57^ Reproduced with permission from ref. 6 and 57. Copyright 2021, Elsevier, and 2020, Wiley, respectively. (C) ADSCs adhesion and proliferation on covalently grafted TiNT-TAN surface after 4 and 7 days,^48^ and osteocalcin expression on TiNT, TiNT(TA/HP), and TiNT(TA/HA) after 1 and 3 weeks.^6^ Reproduced with permission from ref. 48 and 6. Copyright 2025, American Chemical Society, and 2021, Elsevier, respectively. (D) SEM images depicting mineralization and ADSC morphology on TiNT, TiNT(TA/HP), and TiNT(TA/HA) surfaces after 1 and 3 weeks of osteogenic culture.^6^ Reproduced with permission from ref. 6. Copyright 2021, Elsevier. (E) Immunofluorescence and corresponding SEM images of ADSCs after 3 weeks of differentiation on Zn- and Sr-doped TiNT surfaces (left), with associated ALP activity (middle) and osteocalcin area fraction.^34^ Reproduced with permission from ref. 34. Copyright 2024, Royal Society of Chemistry. (F) Representative fluorescence images showing integration of PEM modified surfaces with different cell types: Smooth Muscle Cells (SMCs), Endothelial Cells (ECs), and ADSCs.^6,56^ (G and H) showing the cell adherence and proliferation of SMCs, ECs, respectively.^56^ Reproduced with permission from ref. 6 and 56. Copyright 2021, Elsevier and 2022, Springer Nature. (I) Cell migration and wound healing over PEM modified surfaces with ECs.^56^ Reproduced with permission from ref. 56.

An implanted medical device is like an injury, and, as part of the wound-healing process, stem cells are recruited to adhere, proliferate, and differentiate at the site.^82^ Therefore, cellular interactions and integration with surrounding tissues are fundamental to the long-term success and functionality of medical implants. When an implant is introduced into the body, its surface immediately interacts with proteins from biological fluids, forming a protein layer that influences subsequent cellular responses.^83^ Effective integration requires the implant material to be biocompatible, supporting the adhesion, proliferation, and differentiation of host cells such as fibroblasts, mesenchymal stem cells, and osteoblasts, while minimizing adverse immune reactions or chronic inflammation.^84^ Titania nanotubes, which are the most studied titania nanostructures, have been found to be particularly favorable for osteoblast proliferation.^39,82^ These nanotubes promote not only adhesion but also the growth and differentiation of mesenchymal stem cells.^85^ The stem cells adhered to the titania nanotube surfaces of optimized size, demonstrated elongated morphology, leading to longer filopodia and enhanced osteogenesis.^86^ The results showed that titania nanotubes containing anatase/rutile phases promoted cell communication and proliferation (Fig. 6).^16^

Titania nanotubes coated with tanfloc-based polyelectrolyte multilayers (PEMs), including combinations such as tanfloc/heparin, tanfloc/glycosaminoglycan, and tanfloc/hyaluronic acid, significantly improve the integration of cells with titania surfaces (Fig. 6).^6,48,87^ When TiNTs are modified with tanfloc and glycosaminoglycan, they demonstrate excellent cytocompatibility and support the adhesion and proliferation of human adipose-derived stem cells (ADSCs) after 7 days of culture.^87^ These modified surfaces also enhance the attachment, migration, and proliferation of endothelial cells, which is critical for the successful endothelialization of medical implants. Notably, this modification selectively encourages endothelial cell growth without promoting smooth muscle cell proliferation, making it particularly advantageous for cardiovascular implant applications. Furthermore, applying tanfloc/heparin coating to titania nanotube surfaces results in increased alkaline phosphatase activity, greater mineral deposition, and higher levels of osteocalcin and calcium compared to uncoated nanotubes, indicating improved osteogenic differentiation properties for human ADSCs.^6^ The presence of polyphenol and amine groups in tanfloc may facilitate bone regeneration, while heparin contributes by interacting with signaling proteins essential for bone formation. The polyphenol and amine moieties in TA may promote bone healing. Additionally, HP plays a crucial role due to its interaction with signaling proteins involved in osteogenesis. For the first time, we show that the titania NT can be modified with TA and HP to promote stem cell differentiation. These surfaces may enhance the early-stage osseointegration of implants, thereby reducing the risk of device failure due to aseptic loosening.

Chitosan and heparin-based polyelectrolyte multilayer (PEM) coatings have been utilized to facilitate the delivery of bone morphogenetic protein-2 (BMP-2), a protein recognized for its role in promoting bone formation and cartilage development. These PEM coatings enable the controlled release of BMP-2, thereby improving the osteogenic capabilities of titania nanotube surfaces. When rat bone marrow cells are cultured on these BMP-2 functionalized surfaces, there is a notable increase in osteocalcin expression and calcium accumulation compared to nanotube surfaces without BMP-2 modification.^57^

A parallel approach to mineralization of these titania nanotube surfaces with trace transition metal ions, such as copper and strontium, has also been investigated. Metal-doped nanotubes enhance stem cell differentiation into new bone cells, as confirmed by increased osteocalcin (OCN) and calcium deposition.^7^ This enhanced differentiation is attributed to trace metals serving as important signaling molecules that promote differentiation and reduce the likelihood of osteoclast formation.^34^ Additionally, TiNTs were successfully modified with manganese-containing bioactive glass (BG) *via* pulsed laser deposition to improve their osteogenic properties. Cell toxicity, viability, adhesion, and proliferation studies demonstrate that BG-TiNT is non-toxic and promotes substantial cell attachment and proliferation. Osteogenic activity outcomes also demonstrate that BG-TiNT enhances the osteogenic differentiation of ADSCs, with increased mineral deposition, osteocalcin expression, and calcium concentration compared to TiNT.^65^

## 6. Evaluating biological activity through Co-culture systems

Conventional monoculture systems provide useful initial screening platforms, but they cannot fully reproduce the complexity of the *in vivo* microenvironment. In contrast, co-culture systems allow direct or indirect interactions among multiple cell types and better reflect cell–cell communication, paracrine signaling, matrix remodeling, and immune modulation. For instance, cell–cell co-culture models are essential for recreating physiological communications, such as the bidirectional signaling between endothelial cells and smooth muscle cells required for the integration of cardiovascular implants.

Furthermore, studying the interaction of biomaterials in a host-microbe co-culture model allows researchers to understand the clinical *“race to the surface*” concept, which was established by Gristina in 1987.^88^ In the context of implant surface engineering, these co-culture models are particularly valuable for evaluating the competition between host cells, such as osteoblasts, mesenchymal stem cells, or endothelial cells, and colonizing bacteria. These systems help determine whether a surface promotes not only cell attachment and differentiation but also balanced tissue regeneration and host defense. Because an implant surface *in vivo* is rapidly covered by ECM proteins, followed by immediate competition between surrounding tissue cells and bacteria for surface occupancy.^88,89^ Co-culturing models (both cell–cell and cell–bacteria) provide a much more accurate evaluation of how a biomaterial surface will actually behave upon implantation.

Evaluating biomaterials in a host-microbe co-culture offers a highly translatable model for studying the clinical “race to the surface” in orthopedic implants, where tissue regeneration competes with bacterial infection. To evaluate this coordinated response, our group utilized a co-culture of human ADSCs and *Staphylococcus aureus* on titania nanotubes coated with chitosan/heparin PEM for localized gentamicin delivery.^18^ This co-culture model revealed that while providing gentamicin in a solution effectively reduced bacteria, it resulted in poorly spread, round hADSCs. In contrast, localized delivery from the modified nanotubes effectively inhibited *S. aureus* adhesion while simultaneously allowing the hADSCs to reach the surface first. By day 7 in this co-culture, the stem cells successfully attached and maintained a spread morphology, a necessary precursor to osteogenic differentiation and bone repair.^18^

Beyond host-microbe models, cell–cell co-culture systems are utilized to better mimic the *in vivo* environment of cardiovascular tissue and regulate cell phenotypes by recreating the vital bidirectional signaling between endothelial cells (ECs) and underlying smooth muscle cells (SMCs).^90^ For instance, our group evaluated nanostructured poly(3-caprolactone) (PCL) surfaces modified with collagen using a direct EC/SMC co-culture. While flat PCL surfaces failed to support confluent cell growth, collagen-immobilized PCL nanowires promoted confluent, aligned, and spindle-shaped morphologies for both cell types. Crucially, this co-culture environment enhanced VE-cadherin expression (a protein vital for healthy EC cell–cell junctions) and decreased SMemb expression (a marker of undifferentiated SMCs), indicating that SMCs were successfully guided toward a healthy, contractile phenotype.^90^

Utilizing targeted co-culture approaches complements traditional vitro screening strategies, enabling researchers to ensure that modified surfaces can successfully regulate distinct cell phenotypes, promote balanced tissue regeneration, and protect against clinical complications prior to *in vivo* implantation.

## 7. Summary and future prospects

Biomedical devices and implants are essential components of contemporary healthcare, effectively addressing critical medical needs across diverse populations and regions. Titanium and its alloys are widely favored for the fabrication of modern implants due to their excellent mechanical properties, corrosion resistance, and high biocompatibility, making them particularly suitable for orthopedic, dental, and other applications. Despite these advantages, titanium implants still encounter clinical challenges, including limited osseointegration and vulnerability to bacterial colonization, which compromise long-term success. Surface functionalization has emerged as a pivotal strategy to address these challenges, enabling the optimization of biological interactions and mechanical performance. By modifying surface properties through nanoengineering and subsequent functionalization with bioactive polymers, small molecules, or metal ions, titanium implants demonstrate improved integration with bone tissue, enhanced hemocompatibility, and greater resistance to bacterial infection. This review summarizes significant advancements achieved in our laboratory over recent decades, highlighting how dual surface modification approaches have significantly enhanced the *in vitro* performance of titanium-based implants. These innovations contribute to more reliable osseointegration, reduce the risk of infection, and increase long-term stability, ultimately supporting more successful implantation outcomes.

The development of multifunctional surfaces that combine antibacterial, osteoinductive, and anti-inflammatory properties is particularly promising for extending implant lifespan and improving patient outcomes. These studies underscore the potential of dual-functional surfaces to enhance implant integration and reduce failure risk; however, most findings remain at the proof-of-concept stage and require further validation in clinically relevant models. Future progress in this area will depend on a more critical focus on translational barriers. Key challenges include coating stability under physiological conditions, compatibility with sterilization procedures, reproducibility across fabrication batches, scale-up for manufacturing, long-term *in vivo* performance, and regulatory requirements. In addition, future studies should move beyond conventional monoculture assays and incorporate co-culture systems, organ-on-chip platforms, and other microenvironment-mimicking models to better predict biological performance *in vivo*. These approaches will be important for bridging the gap between laboratory studies and clinical translation, particularly for surfaces designed to simultaneously support tissue integration and suppress infection.

Computational methods may also support this effort by helping prioritize coating parameters, screen candidate materials, and predict surface–cell–bacteria interactions. In practice, artificial intelligence and machine learning can complement experimental work when trained on high-quality, standardized datasets and validated in relevant biological models. Multiscale simulations may further help connect molecular-level surface properties with cellular and tissue responses, thereby improving the rational design of titanium-based implants. Overall, future success will depend on integrating materials engineering, biological validation, and translational strategy to develop implant surfaces that are not only effective *in vitro* but also practical for clinical use.

## Author contributions

Conceptualization: R. S. methodology/outline: R. S, K. C. P. writing – original draft: A. V. S, R. S. writing – review & editing: R. S, K. C. P, A. V. S. supervision: K. C. P and R. S.

## Conflicts of interest

The authors have no conflict of interest.

## Data Availability

No primary research results, software, or code have been included, and no new data were generated or analyzed as part of this review.

## References

[cit1] Dobrzański L. A., Dobrzańska-Danikiewicz A. D., Dobrzański L. B. (2021). Effect of Biomedical Materials in the Implementation of a Long and Healthy Life Policy. Processes.

[cit2] Mastnak T., Maver U., Finšgar M. (2022). Addressing the Needs of the Rapidly Aging Society through the Development of Multifunctional Bioactive Coatings for Orthopedic Applications. Int. J. Mol. Sci..

[cit3] Vayalappil M. C. (2023). Medical implants: evolution during the last hundred years. Opn. Med. Sci. Technol. Health.

[cit4] Savargaonkar A. V., Holloway E., Popat K. C. (2025). Alkali-Treated, Nanostructured-Micro-Porous Titanium Surfaces Enhance Osteogenic Differentiation of Adipose Derived Stem Cells. Appl. Sci..

[cit5] Zhu G., Wang G., Li J. J. (2021). Advances in implant surface modifications to improve osseointegration. Mater. Adv..

[cit6] Sabino R. M., Mondini G., Kipper M. J., Martins A. F., Popat K. C. (2021). Tanfloc/heparin polyelectrolyte multilayers improve osteogenic differentiation of adipose-derived stem cells on titania nanotube surfaces. Carbohydr. Polym..

[cit7] Savargaonkar A. V., Madruga L. C., Munshi A. H., Popat K. C. (2024). Titania nanotubes modified with copper enhance osteogenic differentiation of adipose derived stem cells. RSC Adv..

[cit8] Manivasagam V. K., Popat K. C. (2021). Hydrothermally treated titanium surfaces for enhanced osteogenic differentiation of adipose derived stem cells. Mater. Sci. Eng. C.

[cit9] Chandorkar Y., Ravikumar K., Basu B. (2019). The Foreign Body Response Demystified. ACS Biomater. Sci. Eng..

[cit10] Souza J. G. S., Bertolini M. M., Costa R. C., Nagay B. E., Dongari-Bagtzoglou A., Barão V. A. R. (2021). Targeting implant-associated infections: titanium surface loaded with antimicrobial. iScience.

[cit11] Ashcraft M., Douglass M., Chen Y., Handa H. (2021). Combination strategies for antithrombotic biomaterials: an emerging trend towards hemocompatibility. Biomater. Sci..

[cit12] Rahmati M., Silva E. A., Reseland J. E. A., Heyward C., Haugen H. J. (2020). Biological responses to physicochemical properties of biomaterial surface. Chem. Soc. Rev..

[cit13] Pešáková V., Kubies D., Hulejová H., Himmlová L. (2007). The influence of implant surface properties on cell adhesion and proliferation. J. Mater. Sci. Mater. Med..

[cit14] Savargaonkar A. V., Holloway E., Madruga L. Y. C., Pereira B. L., Soares P., Popat K. C. (2025). Anti-Bacterial Properties and Hemocompatibility of Alkali Treated Nano-Structured Micro-Porous Titanium Surfaces. Biomimetics.

[cit15] Bartlet K., Movafaghi S., Dasi L. P., Kota A. K., Popat K. C. (2018). Antibacterial activity on superhydrophobic titania nanotube arrays. Colloids Surf. B Biointerfaces.

[cit16] Dias-Netipanyj M. F., Sopchenski L., Gradowski T., Elifio-Esposito S., Popat K. C., Soares P. (2020). Crystallinity of TiO2 nanotubes and its effects on fibroblast viability, adhesion, and proliferation. J. Mater. Sci. Mater. Med..

[cit17] Su E. P., Justin D. F., Pratt C. R., Sarin V. K., Nguyen V. S., Oh S. (2018). *et al.*, Effects of titanium nanotubes on the osseointegration, cell differentiation, mineralisation
and antibacterial properties of orthopaedic implant surfaces. Bone Jt. J..

[cit18] Wigmosta T., Popat K., Kipper M. J. (2021). Gentamicin-Releasing Titania Nanotube Surfaces Inhibit Bacteria and Support Adipose-Derived Stem Cell Growth in Cocultures. ACS Appl. Bio Mater..

[cit19] Savargaonkar A. V., Munshi A. H., Soares P., Popat K. C. (2023). Antifouling Behavior of Copper-Modified Titania Nanotube Surfaces. J. Funct. Biomater..

[cit20] Bhattacharjee A., Goodall E., Pereira B. L., Soares P., Popat K. C. (2023). Zinc (Zn) Doping by Hydrothermal and Alkaline Heat-Treatment Methods on Titania Nanotube Arrays for Enhanced Antibacterial Activity. Nanomaterials.

[cit21] Singh R., Madruga Y. C., Savargaonkar A., Martins A. F., Kipper M. J., Popat K. C. (2024). *et al.*, Covalent Grafting of Tanfloc on Titania Nanotube Arrays: An Approach to Mitigate Bacterial Adhesion and Improve the Antibacterial Efficacy of Titanium Implants. Adv. Mater. Interfaces.

[cit22] Sabino R. M., Kauk K., Madruga L. Y. C., Kipper M. J., Martins A. F., Popat K. C. (2020). Enhanced hemocompatibility and antibacterial activity on titania nanotubes with tanfloc/heparin polyelectrolyte multilayers. J. Biomed. Mater. Res., Part A.

[cit23] Chen L., Yan C., Zheng Z. (2018). Functional polymer surfaces for controlling cell behaviors. Mater. Today.

[cit24] Ebrahimi F. and Selvamani R., Introduction to smart and magneto-electro-elastic materials nanostructures, in. Mechanics of Smart Magneto-Electro-Elastic Nanostructures, 2021, pp. 1–22, 10.1016/B978-0-12-819653-3.00001-5

[cit25] Siontorou C. G. , Nikoleli G. P., Nikolelis M. T. and Nikolelis D. P., Challenges and Future Prospects of Nanoadvanced Sensing Technology, Advanced Biosensors for Health Care Applications, 2019, 375–396, 10.1016/B978-0-12-815743-5.00015-9

[cit26] KREUTER J. (2007). Nanoparticles—a historical perspective. Int. J. Pharm..

[cit27] Lemos R., Maia F. R., Reis R. L., Oliveira J. M. (2022). Engineering of Extracellular Matrix-Like Biomaterials at Nano- and Macroscale toward Fabrication of Hierarchical Scaffolds for Bone Tissue Engineering. Adv. NanoBiomed Res..

[cit28] Manivasagam V. K., Popat K. C. (2020). In Vitro Investigation of Hemocompatibility of Hydrothermally Treated Titanium and Titanium Alloy Surfaces. ACS Omega.

[cit29] Montgomerie Z., Popat K. C. (2021). Improved hemocompatibility and reduced bacterial adhesion on superhydrophobic titania nanoflower surfaces. Mater. Sci. Eng. C.

[cit30] Li J., Zhang K., Wu J., Zhang L., Yang P., Tu Q. (2015). *et al.*, Tailoring of the titanium surface by preparing cardiovascular endothelial extracellular matrix layer on the hyaluronic acid micro-pattern for improving biocompatibility. Colloids Surf. B Biointerfaces.

[cit31] Popat K. C., Leoni L., Grimes C. A., Desai T. A. (2007). Influence of engineered titania nanotubular surfaces on bone cells. Biomaterials.

[cit32] Smith B. S., Capellato P., Kelley S., Gonzalez-Juarrero M., Popat K. C. (2013). Reduced in vitro immune response on titania nanotube arrays compared to titanium surface. Biomater. Sci..

[cit33] Damiati L., Eales M. G., Nobbs A. H., Su B., Tsimbouri P. M., Salmeron-Sanchez M. (2018). *et al.*, Impact of surface topography and coating on osteogenesis and bacterial attachment on titanium implants. J. Tissue Eng..

[cit34] Bhattacharjee A., Pereira B., Soares P., Popat K. C. (2024). Titania (TiO 2 ) nanotube surfaces doped with zinc and strontium for improved cell compatibility. Nanoscale.

[cit35] Ruckh T. T., Kumar K., Kipper M. J., Popat K. C. (2010). Osteogenic differentiation of bone marrow stromal cells on poly(ε-caprolactone) nanofiber scaffolds. Acta Biomater..

[cit36] La Flamme K. E., Popat K. C., Leoni L., Markiewicz E., La Tempa T. J., Roman B. B. (2007). *et al.*, Biocompatibility of nanoporous alumina membranes for immunoisolation. Biomaterials.

[cit37] Bechara S., Popat K. C. (2013). Micro-Patterned Nanowire Surfaces Encourage Directional Neural Progenitor Cell Adhesion and Proliferation. J. Biomed. Nanotechnol..

[cit38] Vijay Savargaonkar A., Coutinho Madruga L., Munshi A H., Popat K C. (2024). Titania nanotubes modified with copper enhance osteogenic differentiation of adipose derived stem cells. RSC Adv..

[cit39] Cowden K., Dias-Netipanyj M. F., Popat K. C. (2019). Effects of titania nanotube surfaces on osteogenic differentiation of human adipose-derived stem cells. Nanomedicine.

[cit40] Sorkin J. A., Hughes S., Soares P., Popat K. C. (2015). Titania nanotube arrays as interfaces for neural prostheses. Mater. Sci. Eng. C.

[cit41] Smith B. S., Yoriya S., Grissom L., Grimes C. A., Popat K. C. (2010). Hemocompatibility of titania nanotube arrays. J. Biomed. Mater. Res., Part A.

[cit42] Lin W. T., Zhang Y. Y., Tan H. L., Ao H. Y., Duan Z. L., He G. (2016). *et al.*, Inhibited Bacterial Adhesion and Biofilm Formation on Quaternized Chitosan-Loaded Titania Nanotubes with Various Diameters. Materials.

[cit43] Unal M., Creecy A., Nyman J. S. (2018). The Role of Matrix Composition in the Mechanical Behavior of Bone. Curr. Osteoporos. Rep..

[cit44] Lin X., Patil S., Gao Y. G., Qian A. (2020). The Bone Extracellular Matrix in Bone Formation and Regeneration. Front. Pharmacol.

[cit45] Wang Q., Zhou P., Liu S., Attarilar S., Ma R. L. W., Zhong Y. (2020). *et al.*, Multi-Scale Surface Treatments of Titanium Implants for Rapid Osseointegration: A Review. Nanomaterials.

[cit46] Dec P., Modrzejewski A., Pawlik A. (2023). Existing and Novel Biomaterials for Bone Tissue Engineering. Int. J. Mol. Sci..

[cit47] Zhu L., Luo D., Liu Y. (2020). Effect of the nano/microscale structure of biomaterial scaffolds on bone regeneration. Int. J. Oral Hist..

[cit48] Singh R., Madruga L. Y. C., Savargaonkar A., Martins A. F., Kipper M. J., Popat K. C. (2025). Tanfloc-Modified Titanium Surfaces: Optimizing Blood Coagulant Activity and Stem Cell Compatibility. ACS Biomater. Sci. Eng..

[cit49] Lord M. S., Foss M., Besenbacher F. (2010). Influence of nanoscale surface topography on protein adsorption and cellular response. Nano Today.

[cit50] Hassan L. B., Saadi N. S., Karabacak T. (2025). Hierarchically rough superhydrophobic metal surfaces fabricated by a sandblasting and hot water treatment process. Int. J. Adv. Des. Manuf. Technol..

[cit51] Wu B., Tang Y., Wang K., Zhou X., Xiang L. (2022). Nanostructured Titanium Implant Surface Facilitating Osseointegration from Protein Adsorption to Osteogenesis: The Example of TiO2 NTAs. Int. J. Nanomed..

[cit52] Zarrintaj P., Seidi F., Youssefi Azarfam M., Khodadadi Yazdi M., Erfani A., Barani M. (2023). *et al.*, Biopolymer-based composites for tissue engineering applications: A basis for future opportunities. Compos. B Eng..

[cit53] Kasza K., Gurnani P., Hardie K. R., Cámara M., Alexander C. (2021). Challenges and solutions in polymer drug delivery for bacterial biofilm treatment: A tissue-by-tissue account. Adv. Drug Deliv. Rev..

[cit54] Singh R., Popat K. C. (2024). Enhancing Antibacterial Properties of Titanium Implants through Covalent Conjugation of Self-Assembling Fmoc-Phe-Phe Dipeptide on Titania Nanotubes. ACS Appl. Mater. Interfaces.

[cit55] Baghersad S., Madruga L. Y. C., Martins A. F., Popat K. C., Kipper M. J. (2023). Expanding the Scope of an Amphoteric Condensed Tannin, Tanfloc, for Antibacterial Coatings. J. Funct. Biomater..

[cit56] Sabino R. M., Kipper M. J., Martins A. F., Popat K. C. (2022). Improved in vitro endothelialization on nanostructured titania with tannin/glycosaminoglycan-based polyelectrolyte multilayers. In Vitro Models..

[cit57] Wigmosta T. B., Popat K. C., Kipper M. J. (2021). Bone morphogenetic protein-2 delivery from polyelectrolyte multilayers enhances osteogenic activity on nanostructured titania. J. Biomed. Mater. Res., Part A.

[cit58] Popat K. C., Eltgroth M., LaTempa T. J., Grimes C. A., Desai T. A. (2007). Decreased Staphylococcus epidermis adhesion and increased osteoblast functionality on antibiotic-loaded titania nanotubes. Biomaterials.

[cit59] Manivasagam V. K., Perumal G., Arora H. S., Popat K. C. (2022). Enhanced antibacterial properties on superhydrophobic micro-nano structured titanium surface. J. Biomed. Mater. Res., Part A.

[cit60] Bartlet K., Movafaghi S., Kota A., Popat K. C. (2017). Superhemophobic titania nanotube array surfaces for blood contacting medical devices. RSC Adv..

[cit61] Virk H. S., Popat K. C. (2022). Erythrocyte interaction with titanium nanostructured surfaces. In Vitro Models..

[cit62] Trujillo N. A., Oldinski R. A., Ma H., Bryers J. D., Williams J. D., Popat K. C. (2012). Antibacterial effects of silver-doped hydroxyapatite thin films sputter deposited on titanium. Mater. Sci. Eng. C.

[cit63] Soares P., Dias-Netipanyj M. F., Elifio-Esposito S., Leszczak V., Popat K. (2018). Effects of calcium and phosphorus incorporation on the properties and bioactivity of TiO2 nanotubes. J. Appl. Biomater..

[cit64] Maghsoudi-Ganjeh M., Wang X., Zeng X. (2020). Nanomechanics and Ultrastructure of Bone: A Review. Comput. Model. Eng. Sci..

[cit65] Sabino R. M., Rau J. V., De Bonis A., De Stefanis A., Curcio M., Teghil R. (2021). *et al.*, Manganese-containing bioactive glass enhances osteogenic activity of TiO2 nanotube arrays. Appl. Surf. Sci..

[cit66] Filipović U., Dahmane R. G., Ghannouchi S., Zore A., Bohinc K. (2020). Bacterial adhesion on orthopedic implants. Adv. Colloid Interface Sci..

[cit67] Wang M., Tang T. (2019). Surface treatment strategies to combat implant-related infection from the beginning. J. Orthop. Transl..

[cit68] Cyteval C., Bourdon A. (2012). Imaging orthopedic implant infections. Diagn. Interv. Imaging..

[cit69] Di Somma A. , Moretta A., Canè C., Cirillo A., Duilio A. and Di S. A., Inhibition of Bacterial Biofilm Formation, in Bacterial Biofilms, 2020, 10.5772/INTECHOPEN.90614

[cit70] Butler J., Handy R. D., Upton M., Besinis A. (2023). Review of Antimicrobial Nanocoatings in Medicine and Dentistry: Mechanisms of Action, Biocompatibility Performance, Safety, and Benefits Compared to Antibiotics. ACS Nano.

[cit71] Neto G. L. B., Quinalia T. R. B., de Almeida D. A., Madruga L. Y. C., Souza P. R., Popat K. C. (2025). *et al.*, Surface coating nanoarchitectonics for optimizing cytocompatibility and antimicrobial activity: The impact of hyaluronic acid positioning as the outermost layer. Int. J. Biol. Macromol..

[cit72] Singh R., Popat K. C. (2024). Enhancing Antibacterial Properties of Titanium Implants through Covalent Conjugation of Self-Assembling Fmoc-Phe-Phe Dipeptide on Titania Nanotubes. ACS Appl. Mater. Interfaces.

[cit73] Schnaider L., Brahmachari S., Schmidt N. W., Mensa B., Shaham-Niv S., Bychenko D. (2017). *et al.*, Self-assembling dipeptide antibacterial nanostructures with membrane disrupting activity. Nat. Commun..

[cit74] Singh R., Sharma S., Kautu A., Joshi K. B. (2024). Self-assembling short peptide amphiphiles as versatile delivery agents: a new frontier in antibacterial research. Chem. Commun..

[cit75] Singh R., Kumar Mishra N., Kumar V., Vinayak V., Ballabh Joshi K. (2018). Transition Metal Ion-Mediated Tyrosine-Based Short-Peptide Amphiphile Nanostructures Inhibit Bacterial Growth. ChemBioChem.

[cit76] Apostolidou C. P., Kokotidou C., Platania V., Nikolaou V., Landrou G., Nikoloudakis E. (2024). *et al.*, Antimicrobial Potency of Fmoc-Phe-Phe Dipeptide Hydrogels with Encapsulated Porphyrin Chromophores Is a Promising Alternative in Antimicrobial Resistance. Biomolecules.

[cit77] Manivasagam V. K., Sabino R. M., Kantam P., Popat K. C. (2021). Surface modification strategies to improve titanium hemocompatibility: a comprehensive review. Mater. Adv..

[cit78] Kuzyk P. R. T., Schemitsch E. H. (2011). The basic science of peri-implant bone healing. Indian J Orthop.

[cit79] Sabino R. M., Kauk K., Movafaghi S., Kota A., Popat K. C. (2019). Interaction of Blood Plasma Proteins with Superhemophobic Titania Nanotube Surfaces. Nanomedicine.

[cit80] Manivasagam V. K., Popat K. C. (2023). Improved Hemocompatibility on Superhemophobic Micro–Nano-Structured Titanium Surfaces. Bioengineering.

[cit81] Movafaghi S., Leszczak V., Wang W., Sorkin J. A., Dasi L. P., Popat K. C. (2017). *et al.*, Hemocompatibility of Superhemophobic Titania Surfaces. Adv. Healthc. Mater..

[cit82] Cowden K., Dias-Netipanyj M. F., Popat K. C. (2019). Adhesion and Proliferation of Human Adipose-Derived Stem Cells on Titania Nanotube Surfaces. Regen. Eng. Transl. Med..

[cit83] Meyers S. R., Grinstaff M. W. (2011). Biocompatible and bioactive surface modifications for prolonged in vivo efficacy. Chem. Rev..

[cit84] Huzum B., Puha B., Necoara R. M., Gheorghevici S., Puha G., Filip A. (2021). *et al.*, Biocompatibility assessment of biomaterials used in orthopedic devices: An overview. Exp. Ther. Med..

[cit85] Kong K., Chang Y., Hu Y., Qiao H., Zhao C., Rong K. (2022). *et al.*, TiO2 Nanotubes Promote Osteogenic Differentiation Through Regulation of Yap and Piezo1. Front. Bioeng. Biotechnol..

[cit86] Dias-Netipanyj M. F., Cowden K., Sopchenski L., Cogo S. C., Elifio-Esposito S., Popat K. C. (2019). *et al.*, Effect of crystalline phases of titania nanotube arrays on adipose derived stem cell adhesion and proliferation. Mater. Sci. Eng. C.

[cit87] Da Câmara P. C. F., Balaban R. C., Hedayati M., Popat K. C., Martins A. F., Kipper M. J. (2019). Novel cationic tannin/glycosaminoglycan-based polyelectrolyte multilayers promote stem cells adhesion and proliferation. RSC Adv..

[cit88] Gristina A. G. (1987). Biomaterial-Centered Infection: Microbial Adhesion Versus Tissue Integration. Science.

[cit89] Lee J. H., Wang H., Kaplan J. B., Lee W. Y. (2010). Effects of Staphylococcus epidermidis on osteoblast cell adhesion and viability on a Ti alloy surface in a microfluidic co-culture environment. Acta Biomater..

[cit90] Leszczak V., Popat K. C. (2014). Direct co-culture of endothelial and smooth muscle cells on poly(ε-caprolactone) nanowire surfaces. RSC Adv..

